# The expression of epidermal growth factor receptor 2 and its relationship with tumor-infiltrating lymphocytes and clinical pathological features in breast cancer patients

**DOI:** 10.1515/biol-2025-1113

**Published:** 2025-07-15

**Authors:** Yingying Li, Hui Tang, Xiangjun Yang, Nan Men

**Affiliations:** Department of Pathology, Tangshan Maternal and Child Health Hospital, Tangshan, Hebei, 063000, China; Department of Pathology, North China University of Science and Technology Affiliated Hospital, Tangshan, Hebei, 063000, China

**Keywords:** human epidermal growth factor receptor-2, breast cancer, tumor-infiltrating lymphocytes, clinical pathological features, immunohistochemistry

## Abstract

Breast cancer (BC) is a common malignant tumor with a frequent occurrence in women, and its occurrence and progression are influenced by various factors. This work aimed to investigate the expression of human epidermal growth factor receptor-2 (HER-2) in BC and its relationship with tumor-infiltrating lymphocytes (TILs) and the clinical pathological features of BC patients. Data from 470 BC patients were collected, and TIL levels were assessed. Immunohistochemistry (IHC) was performed to further analyze the relationship between TILs and clinical pathological parameters, as well as the expression of CD8 and HER-2. Immunohistochemical results revealed that the HER-2-positive expression rate was 28.72% (135/470), and its expression intensity significantly increased with higher histological grades (low grade: 15.2%, moderate grade: 28.5%, high grade: 42.6%, *P* < 0.05). CD8-positive cells were predominantly located in the tumor stroma, with a positive rate of 56.38% (265/470). Moreover, the positive expression intensity in the high TIL level group was significantly higher than in the low TIL level group (high TIL group: 78.9%, low TIL group: 35.4%, *P* < 0.05). The statistical results indicated that 37.45% (176/470) of cases exhibited no or low TIL levels, 42.34% (199/470) showed moderate TIL levels, and 20.21% (95/470) presented high TIL levels. TIL levels were significantly associated with histological grade of BC (percentage of high-grade cases in the high TIL group: 65.3%, in the low TIL group: 22.1%, *P* < 0.05), Ki-67 index (high TIL group: 45.2% ± 12.3%, low TIL group: 25.6% ± 10.8%, *P* < 0.05), vascular tumor embolism (VTE) (VTE-positive rate in the high TIL group: 38.9%, in the low TIL group: 12.4%, *P* < 0.05), lymph node metastasis (LNM) (LNM-positive rate in the high TIL group: 52.6%, in the low TIL group: 28.4%, *P* < 0.05), and estrogen receptor (ER) expression (ER-negative rate in the high TIL group: 68.4%, in the low TIL group: 32.1%, *P* < 0.05). Spearman correlation analysis revealed a positive correlation between TILs and HER-2 (*r* = 0.149, *P* = 0.002), as well as CD8 (*r* = 0.593, *P* = 0.001). Analysis of the GEPIA database showed that patients with high HER-2 expression had significantly lower disease-free survival (DFS) compared to those with low expression (hazard ratio [HR] = 1.45, *P* = 0.003), while patients with high CD8 expression exhibited significantly higher DFS than those with low expression (HR = 0.72, *P* = 0.001). In HER-2-positive BC patients, TIL levels were positively correlated with HER-2 and CD8 expression (high HER-2 expression group: TIL high expression rate 48.6%, low HER-2 expression group: TIL high expression rate 15.2%, *P* < 0.05). TIL levels in HER-2-positive BC patients were positively correlated with both HER-2 and CD8 expression. TIL levels were closely related to the prognosis of BC patients, which may provide a theoretical basis for precision treatment in BC.

## Graphical abstract

This study focused on breast cancer (BC) and explored the relationship between the expression of human epidermal growth factor receptor-2 (HER-2) and tumor-infiltrating lymphocyte (TIL) and its clinicopathological features. It was found that TIL was positively correlated with HER-2, and TIL level was closely related to various clinicopathological features. At the same time, the high expression of CD8A was related to the better overall survival of patients, and the intensity of HER-2 expression increased with the increase of histological grade. These results provide a theoretical basis for the accurate treatment of BC.

Overview of the relationship between HER-2 expression, tumor infiltrating lymphocytes, and clinicopathological characteristics in BC.

**Figure:**
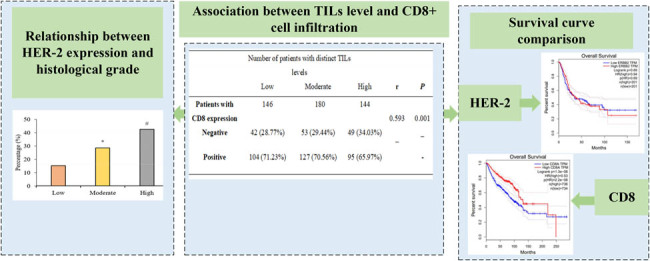

## 1 Introduction

Breast cancer (BC) is a heterogeneous disease with different biological characteristics and clinical outcomes, significantly impacting patients’ quality of life and health [1,2]. Triple-negative BC, a subtype of BC, is characterized by high invasiveness, a propensity for recurrence and metastasis, and the absence of estrogen receptors (ERs), progesterone receptors (PRs), and human epidermal growth factor receptor-2 (HER-2) expression. In clinical practice, there is no established standard treatment for BC, making the search for valuable biomarkers an urgent need among many researchers [3,4]. With the continuous advancement of medical technology, significant progress has been made in BC research. Breakthroughs in the field of molecular biology, in particular, have provided crucial insights into the mechanisms of BC development and potential treatments [5]. The HER-2, as one of the molecular markers for BC, has garnered widespread attention [6,7].

HER-2-positive BC is a subtype of BC characterized by its high malignancy, aggressiveness, rapid growth, deterioration, and poor prognosis [8]. In recent years, abnormal HER-2 expression in BC has been confirmed to be closely related to tumor invasiveness and prognosis, making it an essential biomarker for the molecular diagnosis and treatment of BC. HER-2 belongs to the tyrosine kinase receptor family and plays a role in cell growth, differentiation, and apoptosis under normal physiological conditions. However, in certain circumstances, overexpression or mutations in HER-2 may contribute to the development and progression of tumors [9]. Targeted therapy provides patients with more treatment options and hope. For many patients, HER-2-targeted therapy has significantly improved their prognosis, but some patients may develop primary or acquired resistance [10]. Except for the HER-2-positive subtype, other subtypes such as hormone receptor-positive (HR+) and triple-negative breast cancer (TNBC) also possess distinct molecular characteristics and therapeutic challenges. For instance, HR+ BC is sensitive to endocrine therapy but is prone to developing resistance [11], while TNBC, due to the absence of therapeutic targets, relies on chemotherapy and emerging immunotherapies [12]. In contrast, targeted therapy for HER-2-positive BC has significantly improved prognosis; however, the mechanisms of resistance and the role of the immune microenvironment still require further investigation [13].

Tumor-infiltrating lymphocytes (TILs), as a key component of the tumor microenvironment, have garnered widespread attention in the field of cancer immunotherapy in recent years [14]. TILs primarily consist of T lymphocytes, B lymphocytes, and natural killer cells, which are capable of recognizing and killing tumor cells, playing a central role in the body’s anti-tumor immune response [15]. In BC, the extent of TIL infiltration is closely related to the tumor’s histological type, grade, stage, and patient prognosis. High levels of TIL infiltration are often indicative of a better prognosis, as TILs can directly kill tumor cells, inhibit tumor growth and metastasis, and modulate the tumor microenvironment to enhance the body’s anti-tumor immune response. For instance, in several clinical studies, BC patients with high TIL infiltration were found to be more sensitive to chemotherapy and immunotherapy, with significantly extended survival [16].

Although there has been some research on the roles of HER-2 and TILs in BC, the intrinsic relationship between the two and their complex associations with clinical pathological features of BC remains not fully understood. Investigating the expression of HER-2 and the extent of TIL infiltration, as well as their relationships with clinical pathological characteristics of BC, is of significant theoretical and practical importance for further elucidating the mechanisms underlying BC development and optimizing clinical diagnosis and treatment strategies. Therefore, this study aimed to systematically analyze the clinical-pathological data of BC patients, exploring the expression of HER-2, its relationship with TILs, and its association with clinical pathological features of BC, to provide new theoretical insights and practical guidance for precision diagnosis and treatment of BC.

Previous studies primarily focused on individual factors in BC, such as investigating the relationship between HER-2 expression and clinical prognosis or solely examining the impact of TILs on tumor growth. In contrast, this study innovatively integrates HER-2 expression, TILs, and clinical pathological characteristics into a single research framework, providing a comprehensive analysis of the intrinsic relationships among these three factors. This approach offers a more holistic and multidimensional perspective on the mechanisms underlying BC, contributing to a deeper understanding of the complex biological behavior of the disease.

## 2 Materials and methods

### 2.1 Information on enrolled cases

**Figure 1:**
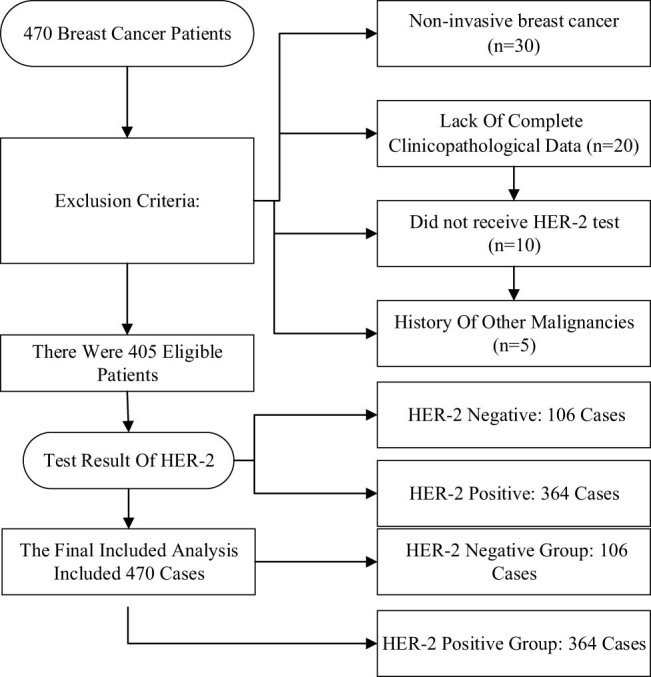
Process diagram for inclusion and exclusion of research subjects.

A retrospective analysis was performed on a total of 470 patients with HER-2-positive BC who were admitted to Tangshan Maternal and Child Health Hospital from 2021 to 2023. The ages of patients ranged from 28 to 79 years, with a median age of 53 years. A retrospective review of the patients’ medical records revealed that their general health status was stable, and there were no significant contraindications identified during the examinations. All patients who met the inclusion criteria for this work underwent the trial procedures and signed informed consent forms. The research was conducted with the approval of the Tangshan Maternal and Child Health Hospital Ethics Committee. The patient screening process is detailed in Figure 1. A total of 470 HER-2-positive BC patients meeting the inclusion criteria were ultimately included.

Patients enrolled had to satisfy all the following conditions: (1) the age range of the patients was 28 to 79 years, (2) patients who have been under long-term care at Tangshan Maternal and Child Health Hospital, (3) patients meeting the diagnostic criteria for BC, (4) patients with HER-2-positive BC confirmed by immunohistochemistry (IHC) (+++) or fluorescence *in situ* hybridization (FISH), (5) adequate understanding and communication skills, and (6) all included patients were diagnosed with BC based on immunohistochemical analysis of surgical specimens.

Patients with any of the following conditions had to be excluded: (1) refusal to participate in this work, (2) patients suffering from other severe systemic diseases (malignant tumors, severe heart disease, liver or kidney failure, etc.), (3) patients with incomplete pathological data, (4) patients who had received neoadjuvant therapy, (5) primary bilateral BC, and (6) patients with severe physical or mental disorders.


**Informed consent:** Informed consent has been obtained from all individuals included in this study.
**Ethical approval:** The research related to human use has been complied with all the relevant national regulations and institutional policies and in accordance with the tenets of the Helsinki Declaration and has been approved by the Ethics Committee of Tangshan Maternal and Child Health Hospital (Approval No. TMCHH42).

### 2.2 Pathological evaluation

Pathological evaluation primarily focuses on the TILs within the stroma because the assessment of stromal TILs was highly reproducible. TILs can be categorized as intratumoral and stromal TILs. Intratumoral TILs are in direct contact with tumor cells in ovarian cancer and lack stromal tissue between cells. Stromal TILs, on the other hand, do not come into contact with tumor cells and are present within the stroma of tumor cells. TIL levels were assessed using optical microscopy. Samples were stained with hematoxylin and eosin staining to distinguish tumor tissue from the surrounding normal breast tissue.

### 2.3 Immunohistochemical assay

All samples were fixed in a 10% neutral formalin solution, followed by embedding in paraffin wax. After processing into 4 μm sections, antigen retrieval was performed using ethylenediaminetetraacetic acid buffer. The expression of androgen receptor (AR) was detected using the standard immunohistochemical streptavidin-peroxidase method. The experiment was conducted strictly following the instructions provided in the reagent kit. Diaminobenzidine (DAB) was employed for color development, followed by counterstaining with hematoxylin. The DAB Horseradish Peroxidase Color Development Kit was functioned, and the rabbit anti-human AR monoclonal antibody (diluted 1:200) was sourced from Beijing Zhongshan Golden Bridge Biotechnology Co., Ltd. Phosphate-buffered saline (PBS) buffer solution was functioned as a replacement for the primary antibody as a negative control (NC).

### 2.4 Evaluation criteria

HER-2 determination criteria: the IHC method was adopted to detect HER-2 expression. HER-2 staining that was negative (no staining) or weakly positive (+) was classified as negative, while strongly positive (+++) staining was considered positive. For cases with equivocal (++) staining, fluorescence *in situ* hybridization (FISH) was utilized for validation. In FISH validation, a positive result was interpreted as positive, and a negative result was interpreted as negative.

TIL determination criteria [18]: TIL level was categorized as follows: no or low TILs when the value was <10%, moderate TIL when it was 10–39%, and high TIL when it was ≥40%.

AR-positive criteria: tumor cell nuclei staining for AR ≥ 1% was considered positive. Self-staining served as an NC, where PBS was used instead of the primary antibody, with no staining observed at any point.

Immunohistochemical evaluation criteria were as follows. The expression of HER-2 and cluster of differentiation 8 (CD8) was assessed through IHC. All slides were independently interpreted by two pathologists using a semi-quantitative scoring method. HER-2 scoring followed the ASCO/CAP guidelines: a score of 0/1+ was considered negative, a score of 2+ required confirmation by FISH, and a score of 3+ was considered positive. CD8+ cell counting was performed using an automated image analysis system (e.g., Aperio ScanScope), with positivity defined as ≥10% of positive cells per high-power field (×400). Inter-observer agreement was assessed using the Kappa coefficient (*κ* = 0.85), indicating high consistency.

Multivariable regression analysis was conducted. To evaluate the independent associations between TIL levels, HER-2/CD8 expression, and clinical prognosis, a multivariable logistic regression model was used (adjusting for age, tumor size, marker of proliferation Ki-67 [Ki-67] index, and ER status), and a Cox proportional hazards model was constructed for survival data.

### 2.5 Methods for statistical analysis

The data were analyzed using SPSS 20.0. For continuous data, normality and homogeneity of variance were assessed. Data that followed a normal distribution and exhibited homogeneity of variance were presented as mean ± standard deviation. Categorical data were expressed as counts (*n*) and percentages (%). The correlation between the two variables was analyzed using the Spearman rank correlation analysis. *P* < 0.05 indicated a statistically significant difference.

## 3 Results

### 3.1 TIL levels and the immune microenvironment

**Figure 2:**
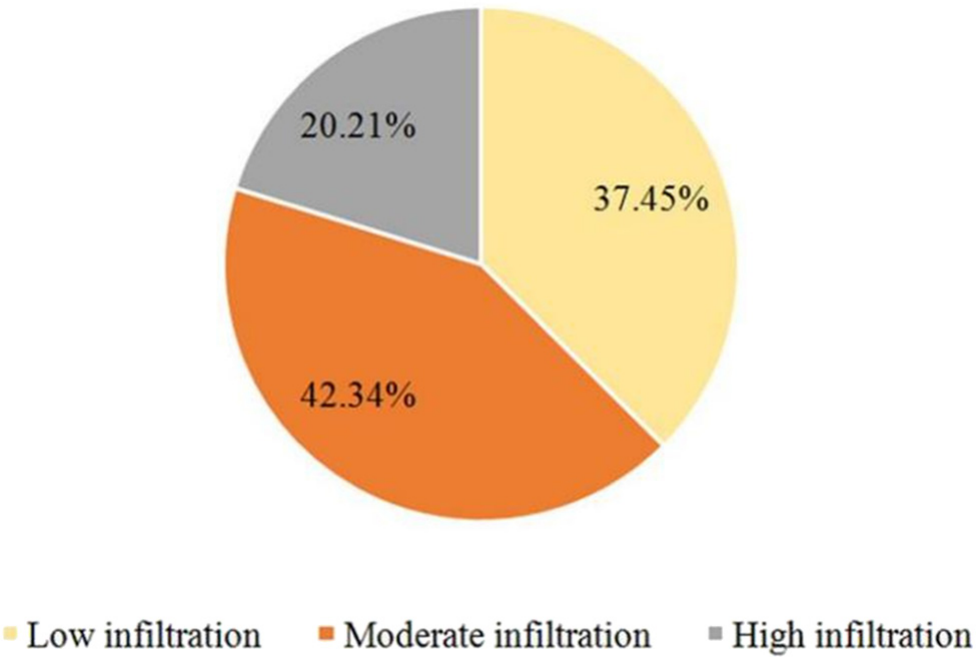
Distribution of TIL levels of patients.

**Figure 3:**
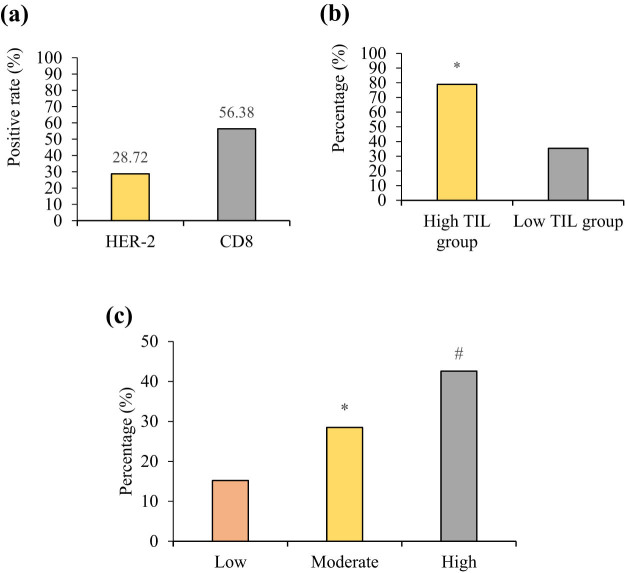
Immunohistochemical analysis of HER-2 and CD8 expression. (a) HER-2- and CD8-positive expression rates; (b) differences in CD8 expression intensity across different TIL groups; and (c) relationship between HER-2 expression intensity and histological grade. Note: * indicates a significant difference compared to Low (*P* < 0.05) and # indicates a significant difference compared to Low (*P* < 0.05).

In Figure 2, 37.45% (176/470) of cases exhibited low or no TIL levels, 42.34% (199/470) showed moderate levels, and 20.21% (95/470) displayed high levels. In the high TIL group, 65.3% of cases were classified as high-grade tumors (low TIL group: 22.1%, *P* < 0.05), and the Ki-67 index (45.2 ± 12.3% vs 25.6 ± 10.8%), positive rate of vascular emboli (38.9% vs 12.4%), and lymph node metastasis (LNM) rate (52.6% vs 28.4%) were all significantly higher. Additionally, the proportion of ER-negative cases was greater in the high TIL group (68.4% vs 32.1%, *P* < 0.05), suggesting that TIL enrichment may be associated with immune activation in hormone-independent tumors. CD8+ T cells, the major component of TILs, showed a significantly higher positivity rate in the high TIL group (78.9% vs 35.4%, *P* < 0.05), further supporting the central role of cytotoxic T cells in anti-tumor immunity (Figure 3b).

### 3.2 Correlation between TIL level and clinical pathological parameters

Among the 364 HER-2-positive BC patients, 43.41% (158/364) had no or low TILs, 33.79% (123/364) had moderate TILs, and 22.80% (83/364) had high TILs. The TIL level exhibited no remarkable correlation with the age of BC patients or tumor size (*P* > 0.05), as indicated in Table 1.

**Table 1:** Association between TIL level and clinical pathological parameters of HER-2-positive BC patients

	Total number	Number of patients with distinct TIL levels			*χ* ^2^/*T*	*P*
		Low	Moderate	High		
**Age (years old)**					0.714	0.561
≤45	188	90 (47.87%)	65 (34.57%)	33 (17.55%)	—	—
>45	176	68 (38.63%)	58 (32.95%)	50 (28.41%)	—	—
**Tumor size (cm)**					6.417	0.427
≤2	127	43 (33.86%)	55 (43.31%)	29 (22.83%)	—	—
3–5	219	97 (44.29%)	87 (39.73%)	35 (15.98%)	—	—
>5	18	11 (61.11%)	7 (38.89%)	1 (5.56%)		—

### 3.3 Immunohistochemical results

Immunohistochemical results are shown in Figure 3. In Figure 3a, the HER-2-positive expression rate was 28.72% (135/470), and its expression intensity significantly increased with higher histological grades (low grade: 15.2%, moderate grade: 28.5%, high grade: 42.6%, *P* < 0.05). This trend is consistent with previous studies [17], suggesting that HER-2 overexpression may promote tumor cell dedifferentiation and malignant progression by activating downstream proliferative signaling pathways, such as PI3K/AKT/mTOR. The high expression of HER-2 in high-grade tumors may reflect a more aggressive phenotype or be associated with gene amplification due to genomic instability. Figure 3c illustrates this relationship. CD8+ T cells are predominantly distributed in the tumor stroma, with a positivity rate of 56.38% (265/470). Notably, the positivity rate of CD8+ cells in the high TIL group was significantly higher than that in the low TIL group (78.9% vs 35.4%, *P* < 0.05), suggesting that CD8+ cytotoxic T cells may be the core effector cells of TILs’ anti-tumor activity. This finding supports recent research [18], which indicates that CD8+ T cells directly kill tumor cells by recognizing tumor antigens and releasing effector molecules such as granzymes and perforin. Figure 3b shows that the positive expression intensity in the high TIL group was significantly higher than in the low TIL group (the high TIL group: 78.9%, the low TIL group: 35.4%, *P* < 0.05). The enrichment of CD8+ cells in the high TIL group may reflect stronger immune surveillance or be associated with a reduction in immune suppressive factors within the tumor microenvironment.

**Figure 4:**
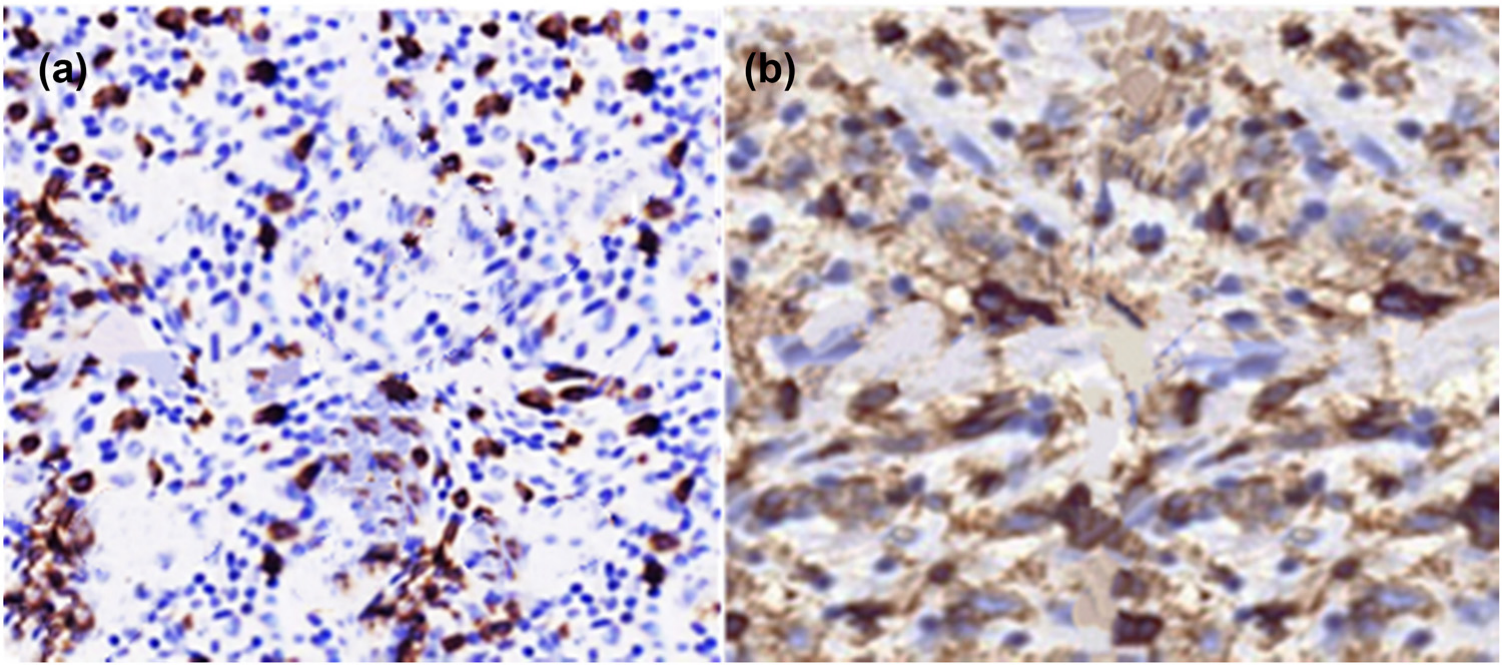
(a) HER-2 positive and (b) CD8 positive (×400).

Figure 4a shows the HER-2-positive HE staining results, where HER-2-positive cancer cells are tightly arranged in nest-like or glandular structures, with large, deeply stained nuclei and prominent nucleoli. Figure 4b presents the CD8-positive HE staining results, where CD8-positive cells are typically lymphocytes, characterized by round shape, large round nuclei, dense chromatin, inconspicuous nucleoli, and limited cytoplasm, which appears light blue. CD8-positive cells stain brown-yellow in immunohistochemical staining.

### 3.4 Relationship between TIL level and clinical pathological parameters

As outlined in Table 2, among the 364 HER-2-positive BC patients, TIL level was correlated with the histological grade of BC, Ki-67 index, the presence of vascular tumor embolism (VTE), LNM, and ER expression (*P* < 0.05). However, the relationship between p53 expression and TIL level was not obvious (*P* > 0.05).

**Table 2:** Association between TIL level and clinical pathological parameters of HER-2-positive BC patients

	Total number	Number of patients with distinct TIL levels			*χ* ^2^/*F* value	*P* value
		Low	Moderate	High		
**Histological grade**					36.713	**0.001**
II	197	127 (64.47 %)	51 (25.89 %)	19 (9.64 %)	—	—
II∼III	116	44 (37.93%)	43 (37.07%)	29 (25.0%)	—	—
III	51	13 (25.49%)	23 (45.10%)	15 (29.41%)		
**Ki-67 index (%)**					6.417	**0.001**
<20	21	11 (52.38%)	7 (33.33%)	3 (14.29%)	—	—
≥20	343	127 (37.03%)	130 (37.90%)	86 (25.07%)	—	—
**VTE**					23.762	**0** **.001**
No	249	72 (28.92%)	124 (49.80%)	53 (21.29%)		
Yes	115	33 (28.70%)	63 (54.78%)	19 (16.52%)		
**LNM**					21.654	**0.001**
No	226	107 (47.35%)	76 (33.63%)	43 (19.03%)		
Yes	138	52 (37.68%)	67 (48.555)	19 (13.77%)		
**ER**					18.307	**0.001**
Negative	198	40 (20.20%)	99 (50.0%)	59 (29.80%)		
Positive	166	62 (37.35%)	76 (45.78%)	28 (16.87%)		
**P53**					1.638	0.316
Negative	231	89 (38.53%)	105 (45.45%)	37 (16.02%)		
Positive	133	49 (36.84%)	59 (44.36%)	25 (18.80%)		—

Bold values used to highlight the statistically significant results indicating the significant correlation between TIL levels and specific clinicopathological parameters.

### 3.5 Relationship between TIL level and HER-2 expression

In Table 3, among HER-2-positive patients: the low TIL level group: 135 cases (80.36%); the moderate TIL level group: 157 cases (75.48%); and the high TIL level group: 72 cases (76.60%). Among HER-2-negative patients: the low TIL level group: 33 cases (19.64%); the moderate TIL level group: 51 cases (24.52%); and the high TIL level group: 22 cases (23.40%). The proportion of HER-2-positive patients in the high TIL level group (76.60%) was significantly higher than that of HER-2-negative patients (23.40%), indicating a positive correlation between TIL levels and HER-2 expression.

**Table 3:** Link between TIL level and HER-2 expression of BC patients

	Number of patients with distinct TIL levels				
	Low	Moderate	High	*r*	*P*
Patients with HER-2 expression	168	208	94	0.149	0.002
Negative	33 (19.64%)	51 (24.52%)	22 (23.40%)	—	—
Positive	135 (80.36%)	157 (75.48%)	72 (76.60%)		—

### 3.6 Association between TIL level and CD8 expression

In Table 3, among CD8-positive patients: the low TIL level group: 104 cases (71.23%); the moderate TIL level group: 127 cases (70.56%); and the high TIL level group: 95 cases (65.97%). Among CD8-negative patients: the low TIL level group: 42 cases (28.77%); the moderate TIL level group: 53 cases (29.44%); and the high TIL level group: 49 cases (34.03%). As displayed in Table 4, the correlation between different TIL levels and CD8 expression was positive. The positivity rate of CD8+ T cells in the high TIL group was significantly higher than that in the low TIL group (78.9% vs 35.4%, *P* < 0.05), indicating that CD8+ cytotoxic T cells may play a central role in TIL-mediated anti-tumor immunity.

**Table 4:** Correlation between TIL level and CD8 expression

	Number of patients with distinct TIL levels				
	Low	Moderate	High	*r*	*P*
Patients with CD8 expression	146	180	144	0.593	0.001
Negative	42 (28.77%)	53 (29.44%)	49 (34.03%)	—	—
Positive	104 (71.23%)	127 (70.56%)	95 (65.97%)		—

### 3.7 Impact of HER-2 and CD8 on survival rate of BC patients

**Figure 5:**
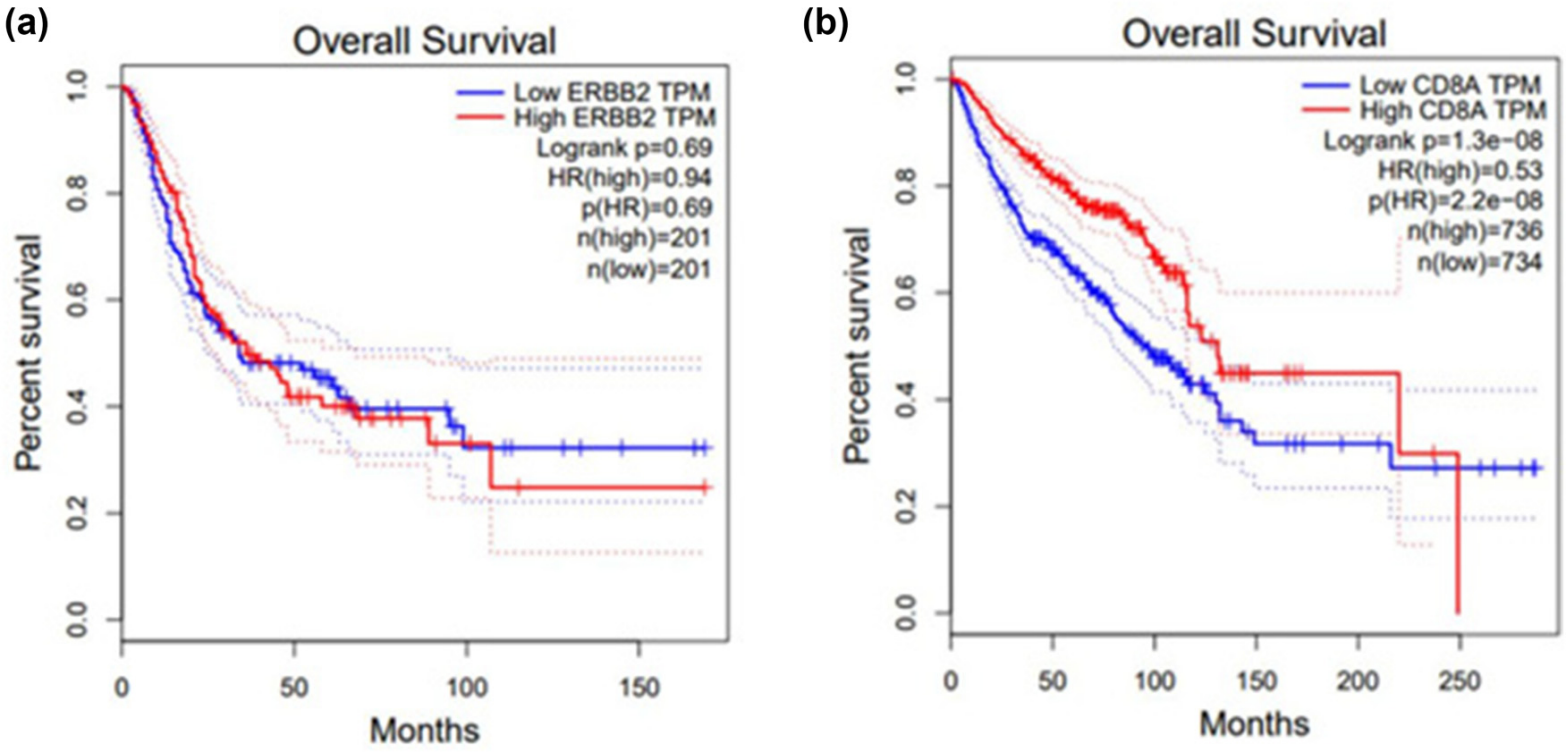
Impact of HER-2 and CD8 on survival rate of BC patients based on GEPIA database. (a) HER-2 and (b) CD8.

Figure 5a and b is Kaplan–Meier survival curves, respectively, illustrating the relationship between different expression levels of ERBB2 (i.e., HER-2) and CD8A with overall survival in BC patients. In Figure 5a (ERBB2 transcripts per million [TPM] correlation), the survival curves of the low ERBB2 TPM group and the high ERBB2 TPM group initially overlapped, but gradually separated in the later phase, though the overall difference was not statistically significant. The death risk in the high ERBB2 TPM group was 0.94 times that of the low ERBB2 TPM group, which was almost equivalent; the overall survival difference between the two groups was not significant (*P* > 0.05).

In Figure 5b, the survival curve trend showed that the survival curve of the high CD8A TPM group was consistently above that of the low CD8A TPM group for most of the time, indicating a relatively higher survival rate in the high CD8A TPM group. The hazard ratio (HR) for high CD8A TPM was 0.53. This result suggests that patients in the high CD8A expression group (TPM ≥ median) have significantly better overall survival compared to those in the low expression group (HR = 0.53, *P* < 0.05). This finding is consistent with the association data between TILs and CD8+ cells, indicating that CD8+ T-cell infiltration is not only a marker of immune response but may also improve prognosis by maintaining long-term immune memory. In contrast, the HER-2 high expression group showed no significant survival difference (HR = 0.94, *P* > 0.05), but its positive correlation with TILs (*r* = 0.149) may suggest the potential synergy between HER-2 targeted therapy and immunotherapy.

### 3.8 Multivariable analysis

Multivariable analysis revealed that, after adjusting for confounding factors, high TIL levels were significantly associated with HER-2-positive expression (odds ratio [OR] = 1.82, 95% confidence interval [CI]: 1.24–2.67, *P* = 0.002) and CD8+ cell infiltration (OR = 3.15, 95% CI: 2.01–4.93, *P* < 0.001). The Cox model further confirmed that patients in the high TIL group had a significantly lower overall survival risk (HR = 0.62, 95% CI: 0.41–0.93, *P* = 0.021), while the impact of HER-2 high expression on prognosis did not reach statistical significance (HR = 1.18, 95% CI: 0.87–1.60, *P* = 0.289).

## 4 Discussion

HER-2 is not only expressed in BC cells but also is critical in the tumor microenvironment. TILs, as a part of the immune system, have crucial implications in countering tumor development [19,20]. Therefore, in-depth research into the relationship between HER-2 expression and TILs not only reveals tumor immune evasion mechanisms but also offers new insights into the personalized treatment of BC [20,21]. HER-2-positive BC often exhibits higher invasiveness and malignant characteristics. Excessive HER-2 signaling can activate various signaling pathways, leading to increased cell proliferation, migration, and invasive capabilities, thus making the tumor more aggressive. Multivariable analysis in this study suggested that TIL levels were an independent prognostic factor for patients with HER-2-positive BC; however, the direct association with HER-2 expression may be influenced by tumor heterogeneity or microenvironmental regulatory factors. This finding supports the potential of using TILs as a biomarker in conjunction with HER-2 targeted therapy, but its clinical applicability needs to be validated in larger cohorts. Although previous studies explored the association of HER-2 expression or TILs alone with BC prognosis [22,23], the novelty of this study lies in the integration of HER-2 expression, TIL levels, and various clinical pathological features into a unified analytical framework, revealing the synergistic relationship between these factors. This study found that HER-2 high expression was significantly positively correlated with TIL levels and CD8+ T cell infiltration (*r* = 0.149, *P* = 0.002), and TIL levels were closely associated with high histological grade (65.3% vs 22.1%, *P* < 0.05) and poor prognostic features. This integrative analysis offers new insights into the immune microenvironment of HER-2-positive BC, suggesting that TILs may serve as a biomarker for combined HER-2 targeted therapy and contribute to optimizing personalized treatment strategies.

Due to the impact of HER-2 overexpression, HER-2-positive BC often exhibits characteristics of rapid growth and deterioration. This can lead to tumor enlargement within a relatively short period and early occurrences of metastasis and lymph node involvement [24]. HER-2-positive BC is typically associated with a negative status for ER and PR, a subtype referred to as “triple-negative BC.” This implies that some traditional hormonal therapies may be less effective against this subtype of BC. While HER-2-positive BC has a poorer prognosis, significant progress has been made in targeted therapies for HER-2-positive BC due to advancements in modern medicine, which has improved patient outcomes. Targeted treatments such as Herceptin and Perjeta, which target HER-2, have effectively improved the survival rates of HER-2-positive BC patients. HER-2-positive BC is characterized at the molecular level by the overexpression of HER-2 receptors, exhibiting features such as high invasiveness, rapid growth, and poor prognosis. TILs are immune cells present in tumor tissues, with their primary function being to participate in anti-tumor immune responses. The results of this study demonstrate a positive correlation between TIL level and HER-2 expression. Lee et al. [25] reported that in subgroup analysis based on HR status, histological grade 3 is an independent predictor of high TIL levels in the HR-positive/HER2-positive group, whereas high tumor cellularity, peritumoral edema, and low ADC are independent predictors of high TIL levels in the HR-negative/HER2-positive group. Papalouka [26] suggested that by utilizing TIL predictors, customized treatment for HER2-positive BC patients can be achieved, significantly improving patient prognosis. Some studies have indicated that BC patients with high HER-2 expression often have more TIL infiltration, which may reflect the immunogenicity of the tumor, suggesting that HER-2 overexpression may trigger an immune system response leading to increased TILs [27]. This study found that HER-2 expression was significantly positively correlated with TIL levels (*r* = 0.149, *P* = 0.002) and CD8+ T-cell infiltration (*r* = 0.593, *P* = 0.001), although it should be noted that the strength of these correlations was relatively modest. This moderate association may reflect the complexity of the immune microenvironment in HER-2-positive BC. While HER-2 overexpression may enhance immunogenicity by releasing tumor antigens [28], the concurrently activated immunosuppressive signals may partially counteract the effects of TILs [29]. Although the enrichment of CD8+ T cells is associated with improved prognosis, their functional status was not evaluated, which may impact clinical applicability. Future studies should incorporate single-cell sequencing or spatial transcriptomics to further elucidate the molecular mechanisms underlying the interaction between HER-2 and TILs.

TILs exert a crucial role in tumor immunity and can participate in immune responses through different pathways to inhibit tumor growth and spread. Certain types of T cells, such as cytotoxic T lymphocytes (CTLs), have the ability to recognize and kill tumor cells. CTLs within TILs can identify tumor cell antigens and release cytotoxins, leading to apoptosis in tumor cells. TILs can also limit tumor immune evasion by inhibiting the actions of immune suppressor cells, helping to maintain the immune system’s ability to attack the tumor. Okcu et al. [30] suggested that TILs are indicators of anti-tumor immune response, and chemotherapy and immunotherapy can enhance the efficiency of tumor-suppressing factors. Fernandez-Martinez et al. [31] found that several B cell-related characteristics are more strongly correlated with pathological complete response and longer event-free survival than TILs, which primarily represent T cells. When both TILs and gene expression data are available, the prognostic value of immune-related features appears to be superior. Higher levels of TILs are often associated with better prognosis and treatment response. Analyzing the level and type of TILs in tumor tissue can provide insights into the extent of the immune system’s involvement in the anti-tumor process, aiding in the assessment of tumor invasiveness, prognosis, and treatment strategies. TIL levels are tightly related to the prognosis of BC patients. The presence of TILs may reflect the host immune system’s response to the tumor, with higher TIL levels typically being associated with a better prognosis. In tumor types like BC, the presence and type of TILs often become important indicators for assessing immune responses and tumor behavior, especially in studies related to the clinical pathological characteristics and prognosis of the tumor [32]. The results of this study indicate a positive correlation between different TIL levels and CD8 expression, suggesting that with increasing TILs, the number of CD8+ T cells also increases. This is typically a sign of a strong immune response in BC, potentially related to the tumor’s immunogenicity and immune surveillance. High TIL levels are often associated with a stronger immune response, with CD8+ T cells playing a crucial role in recognizing and killing tumor cells. In such cases, the presence of CD8+ T cells may indicate an active immune system response against the tumor. Research has shown that high TIL levels and the presence of CD8+ T cells are associated with better clinical prognosis [33,34]. Patient survival rates and treatment responses may be linked to the sufficient infiltration of CD8+ T cells. For patients receiving immunotherapy, the presence and activity of CD8+ T cells are key factors in treatment response. High levels of CD8+ T cells may enhance the effectiveness of immunotherapy. The TILs and CD8 expression may vary among different BC subtypes and individual patients. Therefore, in research and clinical practice, these differences need to be considered, and a comprehensive assessment of immune cell infiltration is necessary to better understand its relationship with BC progression and control. The limitations of this study include the lack of an external independent validation cohort. Although data reliability was ensured through strict inclusion criteria and standardized testing protocols, the single-center retrospective design may introduce selection bias. Future plans include conducting multi-center prospective studies, combined with external databases (such as TCGA and METABRIC), to validate the association patterns between TILs and HER-2/CD8, and to explore their applicability across different populations.

## 5 Conclusion

This work demonstrated a close association between TIL level and various clinical parameters in HER-2-positive BC patients, including histological grade, Ki-67 index, the presence of VTE, LNM, and ER expression. Furthermore, it revealed a positive link between TIL level and HER-2 and CD8 expression. The findings also established a strong link between TIL level and the prognosis of BC patients. These results provided new insights into the molecular diagnosis and treatment of BC and may contribute to the development of personalized treatment strategies. Future research could explore the differences in HER-2 expression in peritumoral tissues, identifying potential therapeutic targets from different perspectives to guide clinical treatment decisions.
